# Jiedu Xiaoliu formula enhances progression-free survival and immune modulation in diffuse large B-cell lymphoma: a retrospective cohort study

**DOI:** 10.3389/fphar.2026.1714532

**Published:** 2026-07-01

**Authors:** Chen Yirong, Zhang Hongyu, Jiang Wenwen, Hu Qi, Lu Jiahui

**Affiliations:** 1 Clinical Hematology Center, Shanghai Municipal Hospital of Traditional Chinese Medicine, Shanghai University of Traditional Chinese Medicine, Shanghai, China; 2 Institute of Hematology, Shanghai Municipal Hospital of Traditional, Chinese Medicine, Shanghai University of Traditional Chinese Medicine, Shanghai, China

**Keywords:** diffuse large B-cell lymphoma, Jiedu Xiaoliu formula, myeloid-derived suppressor cells, progression-free survival, traditional Chinese medicine

## Abstract

**Background:**

Diffuse large B-cell lymphoma (DLBCL) is a clinically heterogeneous subtype of non-Hodgkin lymphoma. While R-CHOP improves prognosis, relapse persists in some patients. Jiedu Xiaoliu Formula (JDXLF), a traditional Chinese medicine (TCM) compound, may synergize with chemotherapy. This study aimed to evaluate the impact of JDXLF’s on progression-free survival (PFS) and immune modulation in DLBCL.

**Methods:**

This retrospective cohort study included 188 patients diagnosed with DLBCL between January 2014 and December 2023 at Shanghai Municipal Hospital of Traditional Chinese Medicine. Patients were stratified into chemotherapy alone (n = 76), chemotherapy + JDXLF (n = 54), and post-chemotherapy JDXLF maintenance (n = 58). Kaplan–Meier survival, LASSO, and multivariate Cox regression were applied to evaluate progression-free survival (PFS) and prognostic factors. Secondary endpoints included overall response rate (ORR), TCM symptom scores, immune biomarkers, and adverse events.

**Results:**

Median PFS was significantly longer in the chemotherapy + JDXLF group (57 months) and JDXLF maintenance group (51 months) compared with chemotherapy alone (39 months; log-rank P < 0.05). Six-month, 1-year, and 2-year PFS rates were consistently higher in both JDXLF groups (all P < 0.05). Cox analysis confirmed that concurrent JDXLF independently reduced the risk of progression (HR = 0.54; 95% CI: 0.30–0.95; P = 0.031). ORR improved with JDXLF (85.2% vs. 76.4% with chemotherapy alone). At 24 months, JDXLF groups showed significantly lower TCM symptom scores (median 5–7 vs. 10; P < 0.001) and reduced monocytic myeloid-derived suppressor cell (M-MDSC) levels (0.08–0.09 vs. 0.15; P < 0.001).

**Conclusion:**

Jiedu Xiaoliu Formula combined with chemotherapy may improve progression-free survival and reduced immunosuppressive M-MDSC levels in DLBCL patients.

## 1 Introduction

Diffuse large B-cell lymphoma (DLBCL) is the most common subtype of non-Hodgkin lymphoma (NHL), accounting for 25%–35% (Xu and Zhao, 2019) of all cases diagnosed annually. It is characterized by significant clinical, biological, and molecular heterogeneity (Allemani et al., 2018; Coiffier et al., 2010), with distinct genetic subtypes (e.g., GCB) exhibiting divergent survival outcomes and therapeutic responses. Despite advancements in immunochemotherapy—especially the incorporation of rituximab into the R-CHOP regimen (rituximab, cyclophosphamide, doxorubicin, vincristine, and prednisone)—certain high-risk subtypes continue to show poor response and high relapse rates (Miyawaki et al., 2022; Coiffier et al., 2002; Sehn et al., 2005; Feugier et al., 2005; Smith, 2017; Lue and O'Connor, 2020), with 5-year survival rates below 50% for patients with refractory disease. He tumor microenvironment, particularly immunosuppressive cells like myeloid-derived suppressor cells (MDSCs), further contributes to treatment resistance. Therefore, improving progression-free survival (PFS) and reducing treatment-related toxicity remain key clinical challenges.

In recent years, traditional Chinese medicine (TCM) has attracted attention for its potential to synergize with conventional therapies by modulating immune checkpoints (e.g., PD-1/PD-L1), inhibiting tumor angiogenesis, and reversing multidrug resistance. Based on long-term clinical practice and pharmacologic studies demonstrating anti-lymphoma properties of key botanical drugs, our team formulated Jiedu Xiaoliu Formula (JDXLF), a compound prescription aimed at detoxifying pathogenic factors and resolving lymphatic masses (Hu et al., 2023). Previous study indicated JDXLF can induce apoptosis in lymphoma cells *in vitro* and *in vivo* (Feng et al., 2025).

However, robust clinical evidence for TCM integration in DLBCL remains scarce, particularly regarding its impact on tumor microenvironment modulation (e.g., myeloid-derived suppressor cells) and long-term survival outcomes. In this study, we retrospectively evaluated the impact of JDXLF on PFS, symptom burden, immune microenvironment, and treatment adverse events in DLBCL patients.

## 2 Methods

### 2.1 Quality assurance and pharmacopoeial compliance

Given that this was a retrospective cohort study using hospital-compounded decoctions rather than a standardized commercial extract, Instead, quality assurance was ensured through the following measures:

- Pharmacopoeial-grade procurement: All botanical drugs were sourced as decoction pieces from the hospital pharmacy of Shanghai Municipal Hospital of Traditional Chinese Medicine. Each incoming batch was accompanied by a batch quality inspection reportissued by supplier, certifying compliance with the Chinese Pharmacopoeia standards for identity (macroscopic character), purity, moisture content, total ash, acid-insoluble ash, extractives, sulfur dioxide residue, heavy metals (lead, cadmium, arsenic, mercury, copper), aflatoxins, and pesticide residues. These reports are retained in the pharmacy archives.
- Patent-documented standardization: The exact composition, weight proportions, and preparation protocol (total dry weight decocted in 3–5-fold water for 30–60 min) are formally disclosed in Chinese authorized invention patent CN 113925944 B, ensuring reproducibility of the formula.

We acknowledge that this level of characterization falls short of the full ConPhyMP ideal for standardized botanical drugal extracts, which we will address in future prospective trials.

### 2.2 Study design and patients

This retrospective cohort study was conducted in the Department of Hematology, Shanghai Municipal Hospital of Traditional Chinese Medicine. A total of 188 patients diagnosed with DLBCL between January 2014 and December 2023 were enrolled. The study was approved by the Institutional Ethics Committee (approval number: 2024SHL-KY-30-01). The informed consent of all the subjects was obtained.

### 2.3 Diagnostic criteria

DLBCL was diagnosed through histopathological examination of lymph node or tissue biopsy specimens, in accordance with the 2022 Chinese Society of Clinical Oncology (CSCO) guidelines (Zhu et al., 2021) and the 2014 Lugano classification system (Cheson et al., 2014) (Supplementary Table S1).

For TCM diagnosis, the criteria for “phlegm-blood stasis binding syndrome” were established following Chinese Hematology of Traditional Chinese Medicine (Chen et al., 2009; Lan et al., 2019) and the 2002 Guidelines for Clinical Research of New Chinese Medicines. Diagnositc requirements included at least two primary and one secondary symptom, combined with tongue and pulse characteristics. Primary symptoms included palpable masses in the neck, axillae, or hypochondrium; abdominal distention; or subcutaneous bruising. Secondary symptoms included emaciation, dark complexion, or hematochezia. Tongue and pulse features were defined as a dark or crimson tongue with greasy yellow coating, accompanied by choppy or rapid pulse.

### 2.4 Inclusion and exclusion criteria

#### 2.4.1 Inclusion criteria

Patients were consecutively enrolled from the institutional database if they met the following pre-specified criteria: ① Histologically confirmed DLBCL according to the WHO classification, with diagnosis verified by lymph node or tissue biopsy, per CSCO Lymphoma Guidelines 2022; ② Completion of ≥6 cycles of standard R-CHOP (rituximab, cyclophosphamide, doxorubicin, vincristine, and prednisone) immunochemotherapy; ③ Age ≥18 years at diagnosis; ④ TCM pattern differentiation consistent with “E’He” (malignant mass) and syndrome differentiation of “phlegm-blood stasis binding” (Medical Administration Department of the State Administration of Traditional Chinese Medicine, 2017); ⑤ Complete medical records, including baseline laboratory tests, imaging studies, and follow-up data; ⑥ Availability for follow-up (in-person, telephone, or medical record review) with informed consent for retrospective data use.

#### 2.4.2 Exclusion criteria

**FIGURE 1:**
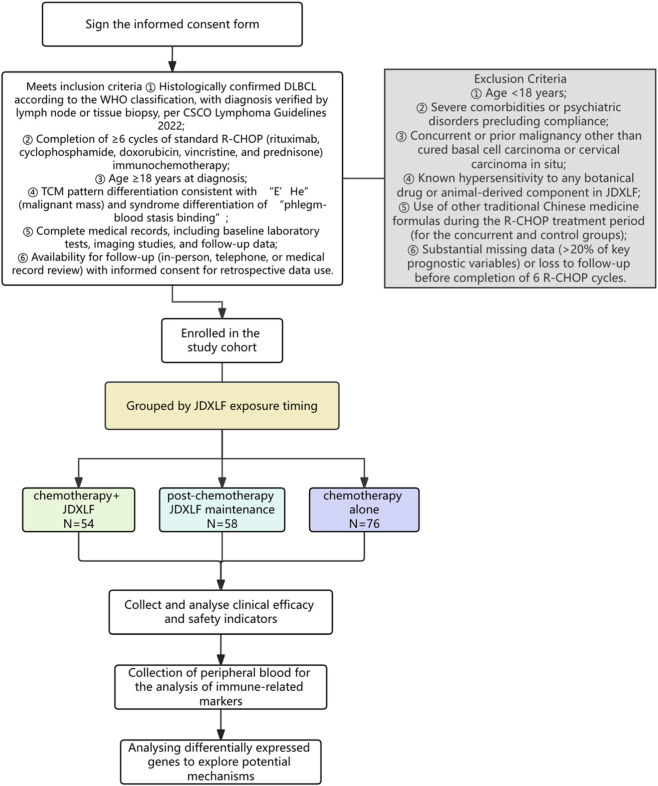
Flow chart of patient enrollment and grouping.

Patients were excluded if they met any of the following: ① Age <18 years; ② Severe comorbidities (e.g., decompensated cirrhosis, end-stage renal disease, NYHA class IV heart failure) or psychiatric disorders precluding compliance; ③ Concurrent or prior malignancy other than cured basal cell carcinoma or cervical carcinoma *in situ*; ④ Known hypersensitivity to any botanical drug or animal-derived metabolite in JDXLF; ⑤ Use of other traditional Chinese medicine formulas during the R-CHOP treatment period (for the concurrent and control groups); ⑥ Substantial missing data (>20% of key prognostic variables) or loss to follow-up before completion of 6 R-CHOP cycles (Figure 1).

### 2.5 Treatment

Based on the application and timing of Jiedu Xiaoliu Formula (JDXLF), patients were categorized into three groups: the Chemotherapy + JDXLF group, which received oral JDXLF (either granules or decoction) concurrently with R-CHOP chemotherapy; the Post-chemotherapy JDXLF maintenance group, in which JDXLF was initiated after completing all chemotherapy cycles; and the Chemotherapy-only group, which received R-CHOP without any TCM intervention. All patients received conventional supportive treatments including hydration, alkalization, and gastric protection.

The Jiedu Xiaoliu Formula was composed of the following botanical drugs per daily dose (Table 1): *Prunella vulgaris* L. \[Lamiaceae; Prunellae Spica] (18g), *Cremastra appendiculata* (D.Don) Makino \[Orchidaceae; Cremastrae Pseudobulbus] (9g), *Salvia chinensis* Benth. \[Lamiaceae; Salviae Chinensis Herba] (15g), *Viola yedoensis* Makino \[Violaceae; Violae Herba] (15g), *Impatiens balsamina* L. \[Balsaminaceae; Impatientis Semen] (6g), *Sparganium stoloniferum* (Buch.-Ham.) Buch.-Ham. ex Graebn. \[Typhaceae; Sparganii Rhizoma] (18g), *Curcuma zedoaria* Val. \[Zingiberaceae; Curcumae Rhizoma] (18g), *Coix lacryma-jobi* L. var. *ma-yuen* (Roman.) Stapf \[Poaceae; Coicis Semen] (30g), *Citrus reticulata* Blanco \[Rutaceae; Citri Reticulatae Pericarpium] (9g), *Pinellia ternata* (Thunb.) Makino \[Araceae; Pinelliae Rhizoma] (9g), *Poria cocos* (Schw.) Wolf \[Polyporaceae; Poria] (18g), *Patrinia villosa* Fisch. ex Trev. \[Caprifoliaceae; Patriniae Herba] (9g), *Carapax Trionycis* Wiegmann \[Trionychidae; Trionycis Carapax] (9g), *Lycium barbarum root bark* L. \[Solanaceae; Lycii Cortex] (18g), *and Glycyrrhiza uralensis* Fisch. \[Fabaceae; Glycyrrhizae Radix et Rhizoma (6g).

**TABLE 1:** Botanical drug composition and relative proportion of the Jiedu Xiaoliu Formula.

Chinese name	Species (Authority) \[Family; Pharmacopoeial drug name]	Part used	Processing	Amount (g per daily dose)	Proportion (% of total)
Xia Ku Cao	Prunella vulgaris L. \[Lamiaceae; Prunellae Spica]	Spike	Dried, raw	18	8.70
Shan Ci Gu	Cremastra appendiculata (D.Don) Makino \[Orchidaceae; Cremastrae Pseudobulbus]	Pseudobulb	Steamed, dried	9	4.35
Shi Jian Chuan	Salvia chinensis Benth. \[Lamiaceae; Salviae chinensis Herba]	Aerial part	Dried, raw	15	7.25
Zi Hua Di Ding	Viola yedoensis Makino \[Violaceae; Violae Herba]	Whole herb	Dried, raw	15	7.25
Ji Xing Zi	Impatiens balsamina L. \[Balsaminaceae; Impatientis Semen]	Seed	Dried, raw	6	2.90
San Leng	Sparganium stoloniferum (Buch.-Ham.) Buch.-Ham. ex Graebn. \[Typhaceae; Sparganii Rhizoma]	Tuber	Vinegar-processed	18	8.70
E Zhu	Curcuma phaeocaulis Val. \[Zingiberaceae; Curcumae Rhizoma]	Rhizome	Vinegar-processed	18	8.70
Sheng Yi Ren	Coix lacryma-jobi L. var. ma-yuen (Roman.) Stapf \[Poaceae; Coicis Semen]	Seed	Dried, raw	30	14.49
Chen Pi	Citrus reticulata Blanco \[Rutaceae; Citri Reticulatae Pericarpium]	Pericarp	Dried, aged	9	4.35
Jiang Ban Xia	Pinellia ternata (Thunb.) Makino \[Araceae; Pinelliae Rhizoma]	Tuber	Ginger-processed	9	4.35
Bai Fu Ling	Poria cocos (Schw.) Wolf \[Polyporaceae; Poria]	Sclerotium	Dried, raw	18	8.70
Bai Jiang Cao	Patrinia scabiosaefolia Fisch. ex Trev. \[Caprifoliaceae; Patriniae Herba]	Whole herb	Dried, raw	9	4.35
Bie Jia	Trionyx sinensis Wiegmann \[Trionychidae; Trionycis Carapax]	Carapace	Vinegar-processed	9	4.35
Di Gu Pi	Lycium barbarum L. \[Solanaceae; Lycii Cortex]	Root bark	Dried, raw	18	8.70
Gan Cao	Glycyrrhiza uralensis Fisch. \[Fabaceae; Glycyrrhizae Radix et Rhizoma]	Root/Rhizome	Honey-processed	6	2.90

The composition of Jiedu Xiaoliu Formula (JDXLF) is a polyherbal decoction comprising 15 botanical drugs and one animal-derived drug. All botanical drugs were procured as pharmacopoeial-grade decoction pieces from the hospital pharmacy of Shanghai Municipal Hospital of Traditional Chinese Medicine. Each incoming batch was accompanied by a batch quality inspection report, certifying compliance with the prevailing edition of the Chinese Pharmacopoeia at the time of procurement. Taxonomic nomenclature was cross-validated using the Medicinal Plant Name Service (MPNS, Royal Botanic Gardens, Kew) and Plants of the World Online (POWO).

The formula was tailored to individual symptoms—for instance, patients presenting with lumbar weakness received additional botanical drugs such as Lycium barbarum, Taxillus chinensis, and Eucommia ulmoides, while those with spontaneous sweating were supplemented with Oryza sativa root, Saposhnikovia divaricata, and *Triticum aestivum*. Cases with pruritus received additions like Kochia scoparia, Dictamnus dasycarpus, and Smilax glabra.

The combined daily dose of dry botanical drugs was placed in a stainless-steel decoction vessel, immersed in 10-fold water at room temperature for 30 min, then simmered for 30 min, filtered through sterile gauze, and the filtrate collected to approximately 400 mL. The decoction was administered orally in two divided doses (approximately 200 mL per dose, twice daily). This standardized preparation protocol was applied uniformly to all patients in this cohort, ensuring batch-to-batch reproducibility within the retrospective study period.

Patients were administered one daily dose divided into two oral portions, with a minimum treatment duration of 6 months. Follow-up assessments were conducted every 2 weeks to monitor treatment adherence and response.

### 2.6 Outcomes and data collection

Primary outcome was progression-free survival (PFS), defined as the time from treatment initiation to disease progression, relapse, or censoring at last follow-up. Secondary outcomes included: (1) Overall Response Rate (ORR) assessed by CT/PET-CT after six cycles of chemotherapy according to NCCN 2016 response criteria (Hu et al., 2023); (2) changes in TCM symptom scores (Supplementary Table S2); (3) laboratory markers including serum cytokines and MDSC subtypes. Liver (ALT, AST), renal (BUN, creatinine) function and gastrointestinal adverse events were collected as safety assessment parameters.

Demographic and Clinical Baseline Characteristics included age, gender, International Prognostic Index (IPI) score, Eastern Cooperative Oncology Group (ECOG) performance status, disease stage (I-II vs. III-IV), pathological subtype (GCB vs. non-GCB), and the presence of B symptoms. B symptoms are a group of systemic (whole-body) symptoms (such as fever, night sweats and weight loss) associated with lymphomas (both Hodgkin and non-Hodgkin lymphomas) and some other cancers.

### 2.7 Statistical analysis

Data analyses were performed using SPSS 25.0 (IBM Corp., Armonk, NY, USA) and R version 4.0.1 (R Foundation for Statistical Computing, Vienna, Austria).

Given the retrospective design without *a priori* power calculation, the stability of the multivariable Cox model was evaluated using the Events Per Variable (EPV) ratio, calculated as the number of PFS events divided by the number of covariates in the final model. An EPV between 5 and 10 was considered indicative of reliable parameter estimation with acceptable bias and variance.

Continuous variables were assessed for normality and presented as mean ± standard deviation, while categorical variables were expressed as numbers (percentages).

Analysis of variance was used for the comparison among continuous variables. Categorical variables were analyzed using chi-square or Fisher’s exact test. Kruskal–Wallis rank-sum tests were applied for nonparametric comparisons.

Survival analysis was performed using the Kaplan-Meier method to calculate the 6-month, 1-year, and 2-year progression-free survival rates of patients in each group. The Kruskal–Wallis rank sum test was used to compare differences in indicators among different groups stratified by PFS time.

Prognostic factors of PFS were selected via Least Absolute Shrinkage and Selection Operator (LASSO) regression and entered into multivariate Cox proportional hazards model. LASSO was not intended for, nor used to construct, a clinical prediction model.

A stratified analysis based on progression-free survival (PFS) duration (<12 months, 12–24 months, >24 months) was also performed to evaluate the association between PFS strata and key outcome measures. A P-value <0.05 was considered statistically significant.

## 3 Results

### 3.1 Baseline characteristics

A total of 188 patients were included: 54 (28.7%) in the chemotherapy + JDXLF group, 58 (30.8%) in the post-chemotherapy JDXLF maintenance group, and 76 (40.4%) in the chemotherapy-only group. Baseline demographic and clinical characteristics, including age, sex, IPI score, ECOG status, disease stage, pathological subtype (GCB vs. non-GCB), and presence of B symptoms, were not significant difference among three groups (*P* > 0.05 for all). There were no significant differences in adverse drug reactions rates across the groups (Table 2).

**TABLE 2:** Baseline characteristics.

Characteristic	Chemotherapy-only, N = 76	Chemotherapy + JDXLF group, N = 54	Post-chemotherapy JDXLF maintenance group, N = 58	P 值
Age	66.5 (58.8, 73.3)	69.0 (61.3, 77.3)	67.0 (60.5, 71.8)	0.221
IPI	2.0 (1.0, 2.3)	2.0 (1.0, 3.0)	2.0 (1.0, 2.0)	0.421
Gender	​	​	​	0.581
Female	39 (51.3%)	30 (55.6%)	35 (60.3%)	​
Male	37 (48.7%)	24 (44.4%)	23 (39.7%)	​
Pathological subtype	​	​	​	0.128
GCB	37 (48.7%)	35 (64.8%)	28 (48.3%)	​
Non-GCB	39 (51.3%)	19 (35.2%)	30 (51.7%)	​
Disease stage	​	​	​	0.507
I ∼ II	35 (46.1%)	22 (40.7%)	30 (51.7%)	​
III ∼ IV	41 (53.9%)	32 (59.3%)	28 (48.3%)	​
ECOG	​	​	​	0.307
0–1	49 (64.5%)	33 (61.1%)	43 (74.1%)	​
2–4	27 (35.5%)	21 (38.9%)	15 (25.9%)	​
B Symptoms	​	​	​	0.173
No	61 (80.3%)	38 (70.4%)	49 (84.5%)	​
Yes	15 (19.7%)	16 (29.6%)	9 (15.5%)	​
Adverse drug reactions	​	​	​	0.818
No	54 (71.1%)	39 (72.2%)	44 (75.9%)	​
Yes	22 (28.9%)	15 (27.8%)	14 (24.1%)	​

### 3.2 Progression-free survival (PFS)

**FIGURE 2:**
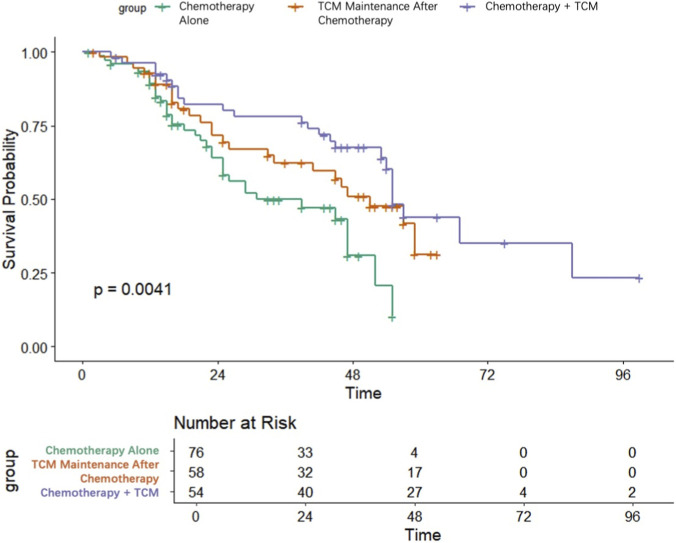
Kaplan–Meier curves showing progression-free survival across treatment groups.

The median PFS (mPFS) for the entire cohort was 51 months. Patients in the chemotherapy + JDXLF group had the longest mPFS (57 months), followed by the post-chemotherapy JDXLF maintenance group (51 months), while the chemotherapy-only group had a shorter mPFS (39 months). The differences between JDXLF groups and chemotherapy-only group were statistically significant (*P* < 0.05). The chemotherapy + JDXLF group showed higher 6-month, 1-year, and 2-year survival rates compared to the chemotherapy-only group (p < 0.05). Patients in post-chemotherapy JDXLF maintenance group exhibited higher 1-year and 2-year survival rates than the chemotherapy-only group (p < 0.05). (Table 3; Figure 2).

**TABLE 3:** PFS by group.

Group	n	mFPS
Total	188	51.0
Chemotherapy + JDXLF group	54	51*
Post-chemotherapy JDXLF maintenance group	58	57*
Chemotherapy-only group	76	39

* Compared with the Chemotherapy-only group:p < 0.05.

### 3.3 LASSO regression analysis

**FIGURE 3:**
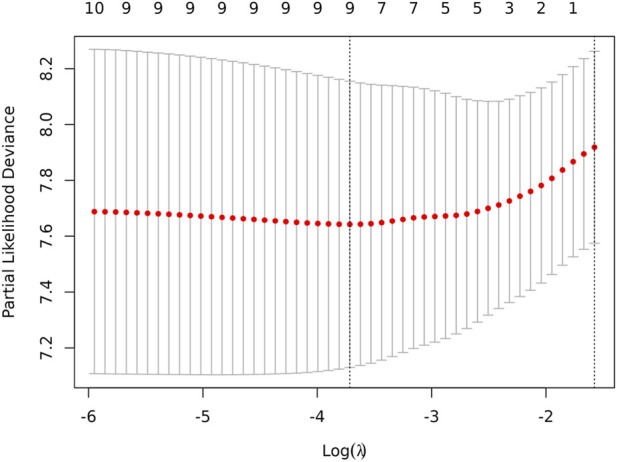
Tuning parameter (λ) selection cross-validation error curve (λ = 0.0243341096455763).

It is important to clarify that LASSO regression was applied here as a variable selection method to reduce dimensionality and prevent overfitting before constructing the multivariable Cox model, not as a predictive model-building tool. LASSO regression with 10-fold cross-validation identified eight potential prognostic variables including treatment group, age, pathological subtype, disease stage, IPI score, ECOG score, presence of B symptoms, and adverse drug reactions.Variance inflation factor [VIF] were <5 for all variables, indicating no significant collinearity (Table 4; Figure 3).

**TABLE 4:** The coefficients of Lasso regression analysis.

Variable	Coefficient
Chemotherapy alone	−0.2089294910
Chemotherapy combined with JDXLF	−0.4103865249
Gender	0.0000000000
Age	−0.0004984945
Pathological subtype	0.7100693352
Disease stage	0.7148945021
IPI (International prognostic Index)	−0.1274543158
ECOG performance status	0.3662496491
B symptoms	0.0310477057
Adverse drug reactions	0.5680101704

### 3.4 Multivariate Cox regression analysis

**FIGURE 4:**
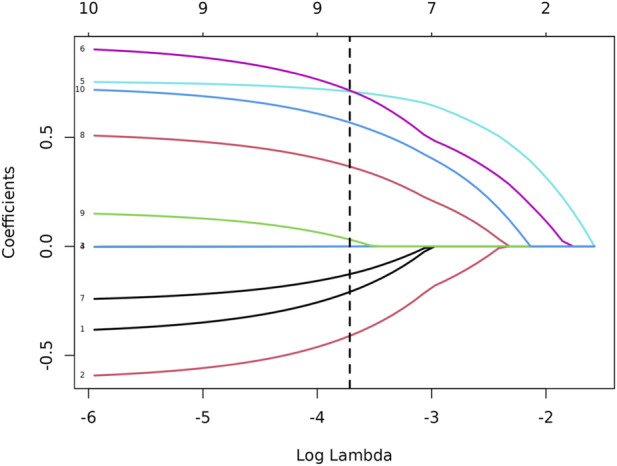
Plot of the Lasso coefficient profiles (λ = 0.0243341096455763).

Multivariate Cox proportional hazards analysis revealed that initiating JDXLF concurrently with chemotherapy was associated with a significantly reduced risk of PFS (HR = 0.54; 95% CI: 0.30–0.95; *P* = 0.031). Pathological subtype (non-GCB vs. GCB: HR = 2.15; 95% CI: 1.35–3.42), advanced disease stage (III–IV vs. I–II: HR = 2.56; 95% CI: 1.50–4.34), ECOG score >2 (HR = 1.71; 95% CI: 1.06–2.74), and the presence of drug-related adverse events (HR = 2.09; 95% CI: 1.29–3.39) were independent prognostic factors of PFS (Table 5; Figure 4).

**TABLE 5:** Cox regression results.

Characteristic	N	HR (Xu and Zhao, 2019)	95% CI (Xu and Zhao, 2019)	p
Group				
Chemotherapy-only group	76	Ref	​	​
Post-chemotherapy JDXLF maintenance group	58	0.66	0.39, 1.12	0.126
Chemotherapy + JDXLF group	54	0.54	0.30, 0.95	0.031
Age	188	1.00	0.98, 1.01	0.745
Pathological subtype				
GCB	100	Ref	​	​
Non-GCB	88	2.15	1.35, 3.42	0.001
Disease stage				
I-II	87	Ref	​	​
III-IV	101	2.56	1.50, 4.34	<0.001
IPI	188	0.77	0.58, 1.02	0.070
ECOG				
0–1	125	Ref	​	​
2–4	63	1.71	1.06, 2.74	0.028
B Symptoms				
No	148	Ref	​	​
Yes	40	1.17	0.68, 2.02	0.574
Adverse drug reactions				
No	137	Ref	​	​
Yes	51	2.09	1.29, 3.39	0.003

### 3.5 Secondary outcomes

The ORR was significantly higher in the chemotherapy + JDXLF group (85.2%) compared with the chemotherapy-only group (76.4%; *P* < 0.05). The ORR in the post-chemotherapy JDXLF maintenance group (75.8%) was comparable to the chemotherapy-only group but significantly lower than the chemotherapy + JDXLF group (*P* < 0.05) (Table 6).

**TABLE 6:** ORR comparison.

Effectiveness	Group (%)		
	Chemotherapy-only group	Chemotherapy + JDXLF group	Post-chemotherapy JDXLF maintenance group
CR	42 (55.3%)	45 (83.3%)	42 (72.4%)
PD	7 (9.2%)	4 (7.4%)	8 (13.8%)
PR	11 (14.5%)	4 (7.4%)	6 (10.3%)
SD	16 (21.1%)	1 (1.9%)	2 (3.4%)
ORR	58 (76.4%)	51 (85.2%)*	47 (75.8%)^#^

* Compared with the Chemotherapy-only group: P < 0.05.

^#^ Compared with the Chemotherapy + JDXLF group: P < 0.05.

At 24 months of PFS, patients in the JDXLF groups had significantly lower TCM symptom scores compared to the chemotherapy-only group. TCM symptom score of the post-chemotherapy JDXLF maintenance group was lower than that of the chemotherapy + JDXLF group, but there was no statistical difference. Symptoms such as emaciation, night sweats, dry mouth, irritability, bitter taste, and chills were more effectively alleviated in the JDXLF groups (*P* < 0.05) (Table 7; Supplementary Table S3). At 24 months PFS, levels of monocytic myeloid-derived suppressor cells (M-MDSC%) were significantly lower in both JDXLF groups compared to chemotherapy alone (*P* < 0.001). The M-MDSC% in the chemotherapy + JDXLF group was significant lower than that in the post-chemotherapy JDXLF maintenance group (*P* < 0.05). The levels of LDH, β2-microglobulin, IL-6, IL-10, IL-4, and G-MDSC% did not differ significantly (Table 8; Supplementary Table S3).

**TABLE 7:** TCM symptom scores.

Characteristic	Chemotherapy-only group N = 33	Chemotherapy + JDXLF group N = 37	Post-chemotherapy JDXLF maintenance group N = 31	p	Test Statistic
Mass/Lump	​	​	​	0.123	​
0	22 (66.7%)	25 (80.6%)	32 (86.5%)	​	​
1	8 (24.2%)	6 (19.4%)	3 (8.1%)	​	​
2	3 (9.1%)	0 (0.0%)	2 (5.4%)	​	​
Fever	​	​	​	0.005	​
0	18 (54.5%)	27 (87.1%)	32 (86.5%)	​	​
1	14 (42.4%)	4 (12.9%)	5 (13.5%)	​	​
2	1 (3.0%)	0 (0.0%)	0 (0.0%)	​	​
Emaciation	​	​	​	0.011	​
0	7 (21.2%)	9 (29.0%)	17 (45.9%)	​	​
1	17 (51.5%)	21 (67.7%)	18 (48.6%)	​	​
2	9 (27.3%)	1 (3.2%)	2 (5.4%)	​	​
Night sweats	​	​	​	0.035	​
0	14 (42.4%)	16 (51.6%)	27 (73.0%)	​	​
1	11 (33.3%)	11 (35.5%)	9 (24.3%)	​	​
2	8 (24.2%)	4 (12.9%)	1 (2.7%)	​	​
Pruritus/Skin Itching	​	​	​	0.335	2.19
0	23 (69.7%)	25 (80.6%)	31 (83.8%)	​	​
1	10 (30.3%)	6 (19.4%)	6 (16.2%)	​	​
Dry mouth and tongue	​	​	​	0.007	​
0	13 (39.4%)	9 (29.0%)	24 (64.9%)	​	​
1	15 (45.5%)	17 (54.8%)	13 (35.1%)	​	​
2	5 (15.2%)	5 (16.1%)	0 (0.0%)	​	​
Fatigue	​	​	​	0.475	​
0	7 (21.2%)	7 (22.6%)	9 (24.3%)	​	​
1	18 (54.5%)	20 (64.5%)	25 (67.6%)	​	​
2	8 (24.2%)	4 (12.9%)	3 (8.1%)	​	​
Sleep (quality)	​	​	​	0.143	​
0	9 (27.3%)	13 (41.9%)	16 (43.2%)	​	​
1	18 (54.5%)	13 (41.9%)	20 (54.1%)	​	​
2	6 (18.2%)	5 (16.1%)	1 (2.7%)	​	​
Mood	​	​	​	0.012	​
0	20 (60.6%)	23 (74.2%)	32 (86.5%)	​	​
1	6 (18.2%)	7 (22.6%)	5 (13.5%)	​	​
2	7 (21.2%)	1 (3.2%)	0 (0.0%)	​	​
Appetite	​	​	​	0.096	​
0	13 (39.4%)	9 (29.0%)	20 (54.1%)	​	​
1	14 (42.4%)	18 (58.1%)	16 (43.2%)	​	​
2	6 (18.2%)	4 (12.9%)	1 (2.7%)	​	​
Heat in Palms and Soles	​	​	​	0.202	3.20
0	22 (66.7%)	25 (80.6%)	31 (83.8%)	​	​
1	11 (33.3%)	6 (19.4%)	6 (16.2%)	​	​
Bitter taste in mouth	​	​	​	0.037	6.61
0	20 (60.6%)	26 (83.9%)	31 (83.8%)	​	​
1	13 (39.4%)	5 (16.1%)	6 (16.2%)	​	​
Palpitations	​	​	​	0.242	​
0	18 (54.5%)	14 (45.2%)	25 (67.6%)	​	​
1	13 (39.4%)	15 (48.4%)	12 (32.4%)	​	​
2	2 (6.1%)	2 (6.5%)	0 (0.0%)	​	​
Cold Intolerance and Cold Limbs	​	​	​	0.037	6.61
0	20 (60.6%)	26 (83.9%)	31 (83.8%)	​	​
1	13 (39.4%)	5 (16.1%)	6 (16.2%)	​	​
Constipation	​	​	​	0.171	​
0	25 (75.8%)	26 (83.9%)	34 (91.9%)	​	​
1	8 (24.2%)	5 (16.1%)	3 (8.1%)	​	​
Oral Ulcers	​	​	​	0.621	​
0	29 (87.9%)	28 (90.3%)	35 (94.6%)	​	​
1	4 (12.1%)	3 (9.7%)	2 (5.4%)	​	​
Pain	​	​	​	0.345	​
0	24 (72.7%)	26 (83.9%)	31 (83.8%)	​	​
1	9 (27.3%)	4 (12.9%)	6 (16.2%)	​	​
2	0 (0.0%)	1 (3.2%)	0 (0.0%)	​	​
Loose Stools	​	​	​	0.226	​
0	29 (87.9%)	30 (96.8%)	31 (83.8%)	​	​
1	4 (12.1%)	1 (3.2%)	6 (16.2%)	​	​
Dizziness	​	​	​	0.733	​
0	31 (93.9%)	29 (93.5%)	36 (97.3%)	​	​
1	2 (6.1%)	2 (6.5%)	1 (2.7%)	​	​
Hemorrhage	​	​	​	0.634	​
0	32 (97.0%)	31 (100.0%)	37 (100.0%)	​	​
1	1 (3.0%)	0 (0.0%)	0 (0.0%)	​	​
Total score	10.0 (8.0, 12.0)	7.0 (5.5, 8.0)	5.0 (4.0, 6.0)	<0.001	41.28

**TABLE 8:** Laboratory markers.

Indicator	Chemotherapy-only group N = 33	Chemotherapy + JDXLF group N = 37	Post-chemotherapy JDXLF maintenance group N = 31	p	H-value
LDH(U/L)	216.00 (176.10, 261.00)	219.80 (179.80, 240.80)	235.40 (198.75, 258.20)	1.37	0.504
β2-MG (mg/L)	2.40 (2.06, 3.14)	2.21 (1.77, 2.84)	2.50 (2.11, 3.09)	2.33	0.312
IL-6 (pg/ml)	4.39 (2.86, 7.25)	2.58 (1.60, 4.41)	2.62 (1.74, 4.37)	7.15	0.028
IL-10 (pg/ml)	5.00 (3.60, 5.00)	5.00 (3.00, 5.00)	5.00 (2.32, 5.00)	1.28	0.527
IL-4 (pg/mL)	6.84 (6.11, 7.42)	6.40 (5.67, 7.10)	6.50 (3.20, 7.36)	1.98	0.372
IL-1β(pg/ml)	5.49 (5.00, 10.50)	5.00 (4.46, 6.08)	5.00 (3.97, 5.00)	5.80	0.055
IFN-r (pg/mL)	12.34 (9.80, 14.21)	12.71 (10.70, 15.70)	12.56 (9.03, 14.30)	0.53	0.767
TNF-a (pg/mL)	10.90 (6.92, 13.40)	8.65 (6.21, 12.50)	9.34 (6.45, 12.70)	1.73	0.420
M-MDSC%	0.15 (0.08, 0.16)	0.08 (0.05, 0.10)	0.09 (0.04, 0.12)	16.33	<0.001
G-MDSC%	0.46 (0.35, 0.60)	0.44 (0.30, 0.66)	0.39 (0.06, 0.62)	0.77	0.680

Stratified analysis by PFS duration (<12 months, 12–24 months, >24 months) showed that longer PFS was associated with significantly lower M-MDSC% and TCM symptom scores in the JDXLF groups (*P* < 0.05). This trend was not observed in the chemotherapy-only group.

No significant differences were observed among the groups in liver enzymes (ALT, AST), renal function (BUN, Cr), or gastrointestinal symptoms (nausea, vomiting, diarrhea), indicating good tolerability of JDXLF when combined with chemotherapy (*P* > 0.05) (Table 9).

**TABLE 9:** Adverse events.

Adverse reaction	​	Chemotherapy-only, N = 76		Chemotherapy + JDXLF group N = 54		Post-chemotherapy JDXLF maintenance group N = 58		Test statistic	P-value
	Indicators	Normal	Abnormal	Normal	Abnormal	Normal	Abnormal	​	​
Liver Function	ALT	74	2	54	0	57	1	2.18	0.34
	AST	75	1	52	2	56	2	0.90	0.64
Renal Function	Cr	72	3	51	3	57	1	0.79	0.68
	BUN	72	4	54	0	57	1	1.15	0.56
Gastrointestinal Tract	Diarrhea	72	4	51	3	56	2	0.92	0.63
	Nausea and vomiting	70	6	53	1	56	2	0.27	0.87

## 4 Discussion

DLBCL is a biologically heterogeneous malignancy with diverse prognostic profiles despite advances in immunochemotherapy. Although R-CHOP remains the standard first-line treatment, a considerable proportion of patients—particularly those with high-risk features—still face relapse or suboptimal therapeutic response. In this study, we demonstrated that the integration of JDXLF with chemotherapy significantly prolonged PFS and improved ORR compared with chemotherapy alone, while simultaneously alleviating TCM-related symptom burden and reducing immunosuppressive M-MDSC levels.

From a TCM perspective, although the term “DLBCL” does not exist in classical Chinese medical texts, its clinical features—such as lymphadenopathy, firm nodules, and progressive course—align with disease entities described as “scrofula (luo li)”, “phlegm nodules (tan he)”, “malignant lumps (e he)”, and “stone carbuncles (shi ju)”. In modern times, the understanding of DLBCL from a TCM perspective has evolved. In 2008, the Hematology Professional Committee of the Chinese Association of Integrative Medicine standardized the nomenclature to “E He,” reflecting the recurrent and aggressive nature of the disease (Piao, 2008). By 2019, a national consensus unified related lymphomas under the designation “E He Bing” (malignant lump disease) (Lan et al., 2019). Etiologically, TCM attributes this condition to deficiency, dampness, phlegm, toxicity, and blood stasis, predominantly affecting the spleen, kidney, and liver, with implications for the heart and lungs. Therapeutic principles emphasize reinforcing vital qi and eliminating pathogenic factors, particularly through liver–kidney regulation and detoxification strategies in advanced disease stages (Xu and Zhou, 2013). These theoretical underpinnings support therapeutic principles centered on reinforcing vital qi and eliminating pathogenic accumulation, which aligns with the pharmacological properties of JDXLF.

Our findings suggest that the timing of JDXLF integration is critical, as patients who received JDXLF concurrently with R-CHOP achieved the most pronounced survival benefit (median PFS 57 months). This supports the concept that early TCM intervention may exert synergistic effects with chemotherapy, possibly by modulating the tumor microenvironment during the most active phase of treatment (Wei et al., 2024). The observation that post-chemotherapy maintenance with JDXLF conferred moderate but less robust benefits (median PFS 51 months) further highlights the importance of early combination rather than delayed initiation.

Immunologically, the reduction of M-MDSCs in JDXLF users is noteworthy. Elevated M-MDSCs are well recognized as a key mechanism of tumor immune evasion and poor prognosis in lymphoma. Our study provides clinical evidence that JDXLF may attenuate this immunosuppressive axis, thereby restoring antitumor immunity (Wei et al., 2024). This is particularly relevant given the increasing interest in immunotherapeutic strategies for DLBCL, such as PD-1/PD-L1 inhibitors, where MDSC-mediated resistance has been reported (McCord et al., 2019; Foureau et al., 2024). Thus, the ability of JDXLF to reduce M-MDSCs could enhance responsiveness not only to chemotherapy but also to future immunotherapy combinations.

Symptom improvement represents another meaningful outcome. Patients in the JDXLF groups experienced significant alleviation of fatigue, night sweats, mood disturbances, and gastrointestinal symptoms compared with chemotherapy alone. This symptomatic relief may reflect both the detoxifying and qi-reinforcing properties of the formula, as well as its potential anti-inflammatory and metabolic effects (Wei et al., 2024). Improved quality of life is particularly relevant for DLBCL patients, as treatment-related toxicity often compromises adherence and long-term outcomes. Importantly, no increase in hepatic, renal, or gastrointestinal toxicities was observed, supporting the safety and tolerability of JDXLF (Zheng et al., 2016; Zhou et al., 2020; Jia and Li, 2007).

We hypothesize that the observed survival benefit may be partially mediated by JDXLF-induced modulation of the tumor immune microenvironment. Specifically, the significant reduction in monocytic myeloid-derived suppressor cells (M-MDSCs) observed in both JDXLF groups at the 24-month landmark (P < 0.001) suggests interference with the IL-6/STAT3-driven expansion of immunosuppressive myeloid populations. Elevated M-MDSCs are recognized as a key mechanism of immune evasion and poor prognosis in lymphoma, and their attenuation may restore anti-tumor immunity and enhance chemosensitivity.

It should be emphasized that no *in vitro* or *in vivo* mechanistic experiments were conducted in the present clinical cohort study. However, preclinical studies underlying JDXLF have been completed and documented in the patent literature (CN 113925944 B). In brief, JDXLF extract (1–8 mg/mL) inhibited proliferation of lymphoma cell lines (Raji [Burkitt lymphoma] and Jeko-1 [mantle cell lymphoma]) in a time- and concentration-dependent manner (IC_50_ ∼4–5 mg/mL at 24 h), induced G2/M cell-cycle arrest, and promoted apoptosis as evidenced by Annexin V-FITC/PI staining and transmission electron microscopy (apoptotic bodies). Mechanistically, JDXLF upregulated pro-apoptotic Bax and Caspase-3 while downregulating anti-apoptotic Bcl-2 at both mRNA (qPCR) and protein (Western blot) levels. Furthermore, in a BALB/c nude-mouse Raji xenograft model, oral administration of JDXLF (low dose 4.25 g/mL; high dose 8.5 g/mL) for 21 days significantly reduced tumor weight and volume (P < 0.05 and P < 0.01, respectively), with immunohistochemical confirmation of increased Bax/Caspase-3 and decreased Bcl-2 expression in tumor tissue. These preclinical data provide a plausible biological rationale for the improved PFS observed in the clinical cohort and support the hypothesis that JDXLF may enhance chemosensitivity by promoting apoptotic cell death.

Although favorable results were obtained in this investigation, a number of limitations cannot be overlooked. First, this is a retrospective, single-center, observational cohort study using existing clinical records; therefore, residual confounding from unmeasured variables (e.g., physician prescribing preference, socioeconomic status, nutritional status) cannot be fully excluded despite the rigorous pre-specified eligibility criteria and demonstrated baseline balance. Second, patient allocation was determined by clinical indication and patient preference during routine practice, not by randomization; consequently, the observed associations are hypothesis-generating rather than definitive proof of causality. Third, the sample size (n = 188) is moderate; while the EPV analysis supports model robustness, the study may be underpowered for detecting smaller effect sizes or subgroup differences. Finally, no *in vitro* or *in vivo* mechanistic experiments were performed in this clinical cohort; however, preclinical data from our prior patent studies (CN 113925944 B) support the biological plausibility of the observed survival benefit. Lastly, mechanistic insights into how JDXLF modulates M-MDSCs or other immune parameters remain speculative and warrant further investigation.

Despite these limitations, the consecutive enrollment, strict eligibility criteria, well-balanced baseline characteristics, and consistent institutional treatment protocols strengthen the internal validity of this real-world evidence. Future multicenter prospective randomized controlled trials are warranted to validate the survival benefit of JDXLF concurrent with R-CHOP and to elucidate its immunomodulatory mechanisms in DLBCL. And a future study will develop and externally validate a JDXLF-specific prognostic nomogram using an independent multicenter cohort.

This retrospective study demonstrates that Jiedu Xiaoliu Formula, when integrated with standard R-CHOP chemotherapy, confers survival and immunological benefits without increasing toxicity. The concurrent use of JDXLF appears to be the optimal strategy, providing both improved clinical outcomes and symptomatic relief. These results suggest that JDXLF could serve as a valuable adjunctive therapy in the management of DLBCL, bridging traditional approaches and modern oncological care.

## 5 Conclusion

Jiedu Xiaoliu Formula, when used in combination with R-CHOP chemotherapy, may improve progression-free survival and modulate immunosuppressive markers in DLBCL patients. Early integration of TCM appears to enhance clinical benefit without compromising safety. These findings support further prospective evaluation of JDXLF as an adjunct therapy in standard lymphoma care.

## Data Availability

The dataset contains sensitive patient information and is subject to privacy restrictions. Due to ethical and legal considerations, the raw data cannot be shared publicly. Requests to access the datasets should be directed to author YC, alesha_chen@163.com.
