# Supplementary material for: Jiedu Xiaoliu formula enhances progression-free survival and immune modulation in diffuse large B-cell lymphoma: a retrospective cohort study
Source: Front Pharmacol. 2026 Jul 1;17:1714532. doi: 10.3389/fphar.2026.1714532 (PMC13368797; doi:10.3389/fphar.2026.1714532)
Supplement: Supplementary file 1 [file Supplementaryfile1.pdf]

Supplementary Table S1. Lymphoma Response Evaluation Criteria (based on Lugano 2014)

| Response Category | Definition |
|---|---|
| **Complete Response (CR)** | • Disappearance of all evidence of disease.• All target and non-target lesions resolved.• Lymph nodes <1.5 cm in longest diameter.• No residual FDG uptake on PET-CT (Deauville score 1–3). |
| **Partial Response (PR)** | • ≥50% decrease in sum of the product of diameters (SPD) of up to 6 target measurable nodes/lesions.• No new lesions.• No increase in non-target lesions.• PET-CT shows residual uptake but reduced compared to baseline. |
| **Stable Disease (SD)** | • Does not meet criteria for CR, PR, or PD.• No significant increase or decrease in disease burden. |
| **Progressive Disease (PD)** | • Appearance of new lesion(s) consistent with lymphoma.• ≥50% increase in SPD of previously involved lesions.• ≥50% increase in a single node/lesion from nadir.• New or recurrent FDG uptake on PET-CT. |

Supplementary Table S2. Traditional Chinese Medicine (TCM) Symptom Score Scale

| Symptom | None (0 Point) | Mild (1 Point) | Moderate (2 Points) | Severe (3 Points) |
|---|---|---|---|---|
| **Mass/Lump** | □ None | □ Involvement of a single site only<br>□ Largest lymph node <30*30 mm<br>□ Splenomegaly <150*60 mm | □ Involvement of multiple sites on the same side of the diaphragm<br>□ Largest lymph node <50*50 mm<br>□ Splenomegaly <180*80 mm | □ Involvement of multiple sites on both sides of the diaphragm<br>□ Largest lymph node >50*50 mm<br>□ Splenomegaly >180*80 mm |
| **Fever** | □ None | □ 37.5-38°C | □ 38-39°C | □ >39°C |
| **Emaciation** | □ None | □ Weight loss of 2kg within one month | □ Weight loss of 2-4kg | □ Weight loss >4kg |

| Symptom | None (0 Point) | Mild (1 Point) | Moderate (2 Points) | Severe (3 Points) |
|---|---|---|---|---|
| **Night Sweats** | □ None | □ Occasional | □ Sweating upon slight activity, presence of night sweats | □ Spontaneous sweating even at rest, profuse night sweats |
| **Skin Itching /Pruritus** | □ None | □ Occasional, mild itching | □ Persistent or intermittent, does not affect sleep | □ Persistent itching, affects sleep |
| **Dry Mouth and Tongue** | □ None | □ Slight dryness in throat, relieved with a little water) | □ Dry throat, relieved by drinking water | □ Severe dry throat, unrelieved by drinking water |
| **Fatigue** | □ None | □ Slight fatigue, does not affect activity | □ Fatigue with reduced activity level | □ Severe fatigue, activity level reduced by half compared to usual |
| **Sleep (Quality)** | □ Normal | □ Early awakening, does not affect work | □ Sleep <4 hours/day, still able to work | □ Complete insomnia, unable to work |
| **Mood** | □ Normal | □ Low mood, relieved by talking | □ Low mood, difficult to self-regulate | □ Low mood, suicidal tendencies |
| **Appetite** | □ Normal | □ Poor, does not affect food intake | □ Food intake reduced by 1/3 compared to usual | □ Food intake reduced by 1/2 compared to usual |
| **Heat in Palms/Soles** | □ None | □ Slight warmth in palms/soles in the evening, | □ Heat in palms/soles, do not want covers, | □ Burning heat in palms/soles, do not want covers, relief from holding cold |

| Symptom | None<br>(0 Point) | Mild<br>(1 Point) | Moderate<br>(2 Points) | Severe<br>(3 Points) |
|---|---|---|---|---|
| | | occasional irritability | occasional irritability | objects, constant irritability |
| **Bitter Taste in Mouth** | □ None | □ Slight bitter taste | □ Bitter taste, food tastes bland | □ Bitter taste, inability to taste food |
| **Palpitations** | □ None | □ Occasional palpitations | □ Frequent palpitations, can resolve spontaneously | □ Severe palpitations, require medication |
| **Cold Intolerance/ Cold Limbs** | □ None | □ Occasional cold hands/feet, unaffected by clothing, occurs with wind exposure | □ Frequent cold limbs, noticeably worse than others, occurs at night | □ Whole body noticeably cold, require clothing for a season warmer than others |
| **Constipatio n** | □ None | □ Dry, hard stools, once daily | □ Hard stools, once every two days | □ Difficulty defecating, once every several days |
| **Oral Ulcers** | □ None | □ Painless ulcers, erythema, mild oral ulcers | □ Painful erythema, edema, ulcers, but able to eat | □ Painful erythema, edema, ulcers, unable to eat |
| **Pain** | □ None | □ Very mild pain, does not affect life or sleep | □ Significant pain, requires pain medication, occasionally wakes from sleep | □ Severe pain, requires pain medication, unable to sleep |
| **Loose Stools** | □ None | □ Loose, unformed stools, once daily | □ Unformed stools, twice daily | □ Unformed stools, three times daily |

| Symptom | None (0 Point) | Mild (1 Point) | Moderate (2 Points) | Severe (3 Points) |
|---|---|---|---|---|
| **Dizziness** | □ None | □ Mild dizziness, occasional, does not affect activity or work | □ Significant dizziness, occurs during activity, relieved by rest | □ Severe dizziness, unsteady gait, persistent, affects activity and work |
| **Hemorrhage/Bleeding** | □ None | □ Occasional gingival bleeding, epistaxis, petechiae/purpura, or bloody stools | □ Recurrent gingival bleeding, epistaxis, petechiae/purpura, bloody stools, or hematemesis in small amounts | □ Gingival bleeding, epistaxis, petechiae/purpura, bloody stools, or hematemesis in large amounts, difficult to stop |

Total Score:________________

Supplementary Table S3. Differences in Metrics by Group and Time Stratum

| Indicator | Chemotherapy-only group, N = 76 | | | | | Post-chemotherapy JDXLF maintenance group, N = 58 | | | | | Chemotherapy + JDXLF group, N = 54 | | | | |
|---|---|---|---|---|---|---|---|---|---|---|---|---|---|---|---|
| | <12Months N = 14 | 12-24Months N = 29 | >24Months N = 33 | Test Statistic | p | <12Months N = 12 | 12-24Months N = 15 | >24Months N = 31 | Test Statistic | p | <12Months N = 6 | 12-24Months N = 11 | >24Months N = 37 | Test Statistic | p |
| **TCM Syndrome Score** | 11.50 (10.25, 12.75) | 10.00 (6.00, 12.00) | 10.00 (8.00, 12.00) | 3.02 | 0.221 | 13.0 (10.3, 13.3) | 11.0 (8.0, 12.0) | 7.0 (5.5, 8.0) | 16.82 | <0.001 | 11.00 (8.50, 12.75) | 6.0 (5.0, 10.5) | 5.0 (4.0, 6.0) | 14.71 | <0.001 |
| **LDH(U/L)** | 205 (150, 274) | 208 (179, 419) | 216 (176, 261) | 1.31 | 0.521 | 200 (181, 241) | 234 (189, 340) | 235 (199, 258) | 2.58 | 0.276 | 199 (128, 267) | 205 (175, 292) | 220 (180, 241) | 0.34 | 0.845 |
| **β2-MG(mg/L)** | 2.32 (1.77, 2.90) | 2.41 (2.05, 3.91) | 2.40 (2.06, 3.14) | 0.74 | 0.690 | 2.38 (2.09, 3.78) | 2.32 (2.11, 3.64) | 2.50 (2.11, 3.09) | 0.25 | 0.881 | 2.97 (2.65, 3.95) | 2.57 (2.33, 3.20) | 2.21 (1.77, 2.84) | 6.10 | 0.047 |
| **IL-6（pg/ml）** | 3.4 (1.7, 5.8) | 4.1 (2.1, 7.7) | 4.4 (2.9, 7.3) | 0.51 | 0.774 | 3.3 (2.1, 7.4) | 3.3 (2.1, 4.5) | 2.6 (1.7, 4.4) | 1.22 | 0.544 | 3.7 (1.9, 5.3) | 5.0 (2.9, 6.9) | 2.6 (1.6, 4.4) | 3.03 | 0.220 |
| **IL-10（pg/ml）** | 5.00 (3.19, 5.00) | 5.00 (5.00, 5.00) | 5.00 (3.60, 5.00) | 1.83 | 0.401 | 5 (4, 5) | 5 (5, 9) | 5 (2, 5) | 4.02 | 0.134 | 5.00 (3.81, 5.00) | 5.00 (3.38, 5.00) | 5.00 (3.00, 5.00) | 0.03 | 0.986 |
| **IL-4(pg/mL )** | 6.41 (6.21, 7.10) | 6.84 (6.28, 7.16) | 6.84 (6.11, 7.42) | 1.36 | 0.507 | 7.17 (6.43, 8.07) | 6.76 (5.89, 7.33) | 6.50 (3.20, 7.36) | 2.94 | 0.230 | 6.68 (5.88, 7.76) | 6.22 (4.17, 7.13) | 6.40 (5.67, 7.10) | 0.84 | 0.658 |
| **IL-1β（pg/ml）** | 5.0 (4.0, 6.3) | 5.0 (5.0, 8.8) | 5.5 (5.0, 10.5) | 2.44 | 0.295 | 5.0 (3.1, 6.1) | 5.0 (3.7, 5.0) | 5.0 (4.0, 5.0) | 0.33 | 0.847 | 7.0 (5.0, 13.9) | 5.0 (5.0, 6.8) | 5.0 (4.5, 6.1) | 3.01 | 0.223 |

| | | | | | | | | | | | | | | | |
|---|---|---|---|---|---|---|---|---|---|---|---|---|---|---|---|
| **IFN-r(pg/mL)** | 12.8 (11.3, 14.7) | 12.1 (10.8, 15.4) | 12.3 (9.8, 14.2) | 1.55 | 0.461 | 12.0 (9.5, 13.7) | 12.9 (10.5, 13.9) | 12.6 (9.0, 14.3) | 0.32 | 0.850 | 11.2 (9.8, 12.2) | 13.4 (12.0, 14.2) | 12.7 (10.7, 15.7) | 2.22 | 0.330 |
| **TNF-a(pg/ml)** | 9 (7, 12) | 8 (5, 19) | 11 (7, 13) | 1.00 | 0.607 | 10 (6, 16) | 8 (6, 20) | 9 (6, 13) | 0.14 | 0.930 | 9 (6, 11) | 11 (7, 13) | 9 (6, 13) | 0.68 | 0.713 |
| **M-MDSC %** | 0.15 (0.14, 0.15) | 0.14 (0.03, 0.16) | 0.15 (0.08, 0.16) | 0.88 | 0.645 | 0.14 (0.12, 0.16) | 0.10 (0.01, 0.12) | 0.09 (0.04, 0.12) | 8.44 | 0.015 | 0.15 (0.12, 0.15) | 0.09 (0.07, 0.10) | 0.08 (0.05, 0.10) | 10.56 | 0.005 |
| **G-MDSC %** | 0.48 (0.39, 0.60) | 0.50 (0.35, 0.62) | 0.46 (0.35, 0.60) | 0.77 | 0.679 | 0.42 (0.36, 0.58) | 0.28 (0.07, 0.40) | 0.39 (0.06, 0.62) | 2.90 | 0.234 | 0.51 (0.41, 0.72) | 0.46 (0.27, 0.55) | 0.44 (0.30, 0.66) | 0.91 | 0.635 |

[1]Median (IQR)

[2]Kruskal-Wallis rank sum
